# Generic substitution of epinephrine autoinjectors: Patient and caregiver perceptions and attitudes

**DOI:** 10.1016/j.jacig.2023.100170

**Published:** 2023-09-14

**Authors:** Sarah E. Ray, Vanessa Boudewyns, Olumurewa Oguntimein, Denise Conti, Raahina Malik, Ila Srivastava, Katharine B. Feibus

**Affiliations:** a RTI International, Research Triangle Park, NC; b Center for Drug Evaluation and Research, US Food and Drug Administration, Silver Spring, Md

**Keywords:** Generic substitution, complex, drug–device combination product, emergency use, patients, caregivers, autoinjector, epinephrine, anaphylaxis, perceptions, attitudes

## Abstract

**Background:**

It is important to understand and address patient and caregiver perceptions about and attitudes toward generic substitution of drug–device combination products.

**Objective:**

The goal of this study was to explore how differences in design and usability features of epinephrine autoinjectors (EAIs) affect patients’ and caregivers’ views of product quality, efficacy, and device usability in an emergency setting and to better understand attitudes about and perceptions of EAI substitution.

**Methods:**

This qualitative, enhanced focus group study was conducted in the United States among adult and adolescent EAI users. A journey mapping exercise was used to explore patient and caregiver perceptions of and barriers to switching to a generic EAI. Discussion topics also focused on questions participants would ask, challenges they would face, and actions they would take if they were switched to a generic EAI.

**Results:**

While patients and caregivers were interested in the potential cost savings and increased access to treatment offered by generic EAIs, they wanted to be informed by their prescriber or pharmacist about generic substitution before or when it occurs. In terms of design differences, participant feedback most often related to differences in look and feel (eg, size, force to execute user tasks, hand grip) or functionality and design differences (eg, the generic version has a twist-off cap at the needle end, and the brand-name EpiPen does not).

**Conclusion:**

Outcomes from these focus groups suggest opportunities for the US Food and Drug Administration, health care professional organizations, and patient advocacy organizations to improve generic drug literacy among adults, adolescents, and health care providers.

Anaphylaxis is an acute, potentially life-threatening systemic allergic reaction that can result in cardiovascular and/or respiratory compromise and death. Prompt recognition and diagnosis of anaphylaxis are essential, and there is international agreement that first-line treatment for anaphylaxis and suspected anaphylaxis is epinephrine 1 mg/mL solution immediately administered intramuscularly into the mid outer thigh, regardless of the severity of initial symptoms.^1^∗ The Joint Task Force on Practice Parameters† made the *strong* recommendation that individuals with a history of anaphylaxis/severe allergic reactions always carry 2 epinephrine autoinjectors (EAIs) at all times.^2^ Despite this, studies have revealed that only about half of patients regularly carry an unexpired EAI.^3^ Availability of and access to high-quality generic versions of US Food and Drug Administration (FDA)-approved EAIs, paired with patient/caregiver confidence in their ability to use a generic EAI, could improve patient and caregiver adherence with clinical best practices.

A generic drug is expected to have the same safety profile and clinical effect as its name-brand counterpart, known as a reference listed drug (RLD). For this reason, generic drugs can be substituted for RLDs. Increased availability of generic versions of EAIs and other drug–device combination products (DDCPs)^4^‡ can result in significant cost savings to patients,§^5^ improved access to treatment, and enhanced treatment adherence.^6^ Regulatory requirements for reference-listed drugs (ie, name-brand drugs) and its generics are listed in Table I.

**Table I:** Regulatory requirements for RLDs and their generics

RLD (name brand)	Generic drug for RLD
Approved through NDA	Approved through an ANDA.
Demonstrates efficacy and safety before approval	An ANDA may not be submitted if clinical investigations are necessary to establish the safety and effectiveness of the proposed drug product. An ANDA relies on the FDA’s finding that the RLD is safe and effective. A drug product approved in an ANDA under section 505(j) of the federal Food, Drug, and Cosmetic Act has demonstrated: (1) that it is the same as the RLD with respect to the active ingredients, conditions of use, route of administration, dosage form, strength, and labeling (with certain permissible differences); and (2) bioequivalence to the RLD. It must also have met the same standards for quality and performance. It is presumed to be therapeutically equivalent to its RLD. Products classified as therapeutically equivalent can be substituted with the full expectation that the substituted product will produce the same clinical effect and safety profile as the prescribed product when administered to patients under the conditions specified in the labeling.
Dosage form	Same as brand drug product.
Drug strengths	Same as brand drug product (may be a subset of brand product strengths).
Route of administration	Same as brand drug product.
Intended use (indications)	Same as brand drug product.∗
Quality and performance standards	Same as brand drug product.
Overall risk profile	Same as brand drug product. If, after approval, new information shows that the overall risk profile is not the same, then the generic product is no longer considered therapeutically equivalent to the brand product and cannot be substituted.
Prescribing information and patient information	Same as the brand product except for differences allowed by regulation because the drug is produced or distributed by different manufacturers; such differences may include differences in expiration date, formulation, bioavailability, or pharmacokinetics; labeling revisions made to comply with current FDA labeling guidelines or other guidance; or omission of an indication or other aspect of labeling protected by patent or accorded exclusivity.
Drug–device combination product	Before approval, the generic drug developer and the FDA review team compare the user interface† design and use the process of the proposed generic product to that of the brand product to ensure that a patient, caregiver, or health care provider will understand how to use the generic product *without additional training* when generic substitution occurs.

*ANDA,* Abbreviated new drug application; *NDA*, new drug application.

∗ Some conditions of use approved for the RLD may be omitted from ANDA labeling because of patents or exclusivity.

† The user interface of a DDCP refers to all components of the combination product with which a user interacts. This includes drug delivery devices, any associated controls and displays, and product labeling and packaging.

Currently, the FDA has approved 3 name-brand (hereafter brand) EAIs—EpiPen (approved in 1987), Adrenaclick (approved in 2009), and Auvi-Q (approved in 2012)—and in 2018 also approved a generic for EpiPen from Teva Pharmaceuticals.^7^ In addition, both EpiPen and Adrenaclick have authorized generics—that is, the brand product labeled with a different name (eg, “epinephrine autoinjector”) and sometimes a different manufacturer name.^8^

Despite extensive use and savings attributed to generic drugs, American consumers and patients have previously expressed misconceptions and uncertainty about generic drugs and their substitution for brand drugs.¶^9^, ^10^, ^11^ While these concerns have decreased over the past 20 years, some mistrust remains.^12^ A 2018 FDA-funded cross-sectional survey of 100 patient and caregiver opinions found that 93% of participants agreed or strongly agreed that generic drugs are as safe and effective as brand drugs, 91% preferred to use generic drugs because they cost less, yet 20% remained unsure about the benefits and risks of generic drugs compared to brand drugs, and 15% remained unsure about which benefits and risks mattered the most to them.^13^

With the increasing role played by complex DDCPs in the acute treatment of disease (eg, drugs provided in single-use or multiple-use pen injectors) and the high cost of brand DDCPs, it is important to understand and address patient and caregiver perceptions about and attitudes toward generic substitution of these complex DDCPs. Our literature search found that only a few published studies have assessed patients’ perceptions of generic substitution of complex DDCPs, especially those used in emergency treatment situations.

This qualitative, enhanced focus group study was conducted in the United States among adult and adolescent EpiPen users (patients and their caregivers) to advance the scientific understanding of patient and caregiver attitudes about and perceptions of generic EAI substitution. The study design focused on learning how differences in design and usability features affect patients’ views of product quality, efficacy, and device usability in an emergency setting.

## Methods

### Study design

Four in-person focus groups took place in the United States during October 2019 and February 2020. Owing to the coronavirus disease 2019 pandemic, we stopped in-person data collection in March 2020 and conducted the remaining 4 focus groups virtually. The virtual focus groups took place on the Zoom web-conferencing platform in June 2022. Our planned number of focus groups was informed by prior research and experience related to theme saturation.^14^

### Participants

To understand the impact of switching to a generic EAI across a variety of patient populations, we included separate groups for adult caregivers of EpiPen users, adult EpiPen users, and adolescent EpiPen users aged 12 to 17 years. More information about eligibility criteria is provided in Table E1, available in the Online Repository at www.jaci-global.org.

### Focus group sessions

Before (in person) or at the start of (virtual) each focus group, participants completed a questionnaire asking about their experience with their current brand of EAI. This included a question about how long they had used an EAI but not how many times they had used one. For all groups, the moderator facilitated the discussion using a semistructured interview guide. To orient the discussion, the moderator first asked questions about participant perceptions of generic drugs to better understand their overall feedback about (and reactions to) generic drugs.

Next, the moderator presented a journey-mapping exercise that presented a potential real-life journey salient to each participant: their brand EAI is unexpectedly replaced with a generic EAI at the pharmacy. The moderator walked participants through a 3-step scenario to project participants’ attitudes and opinions about generic devices in general and generic substitution of their EAI in particular:^15^, ^16^, ^17^

• Step 1. Ordering a refill for their prescription brand EAI.
• Step 2. Picking up the brand EAI and finding out it had been switched to a generic EAI.
• Step 3. Using the generic EAI for the first time.

More information is available in Tables E1 and E2, both available in the Online Repository at www.jaci-global.org.

**Fig 1:**
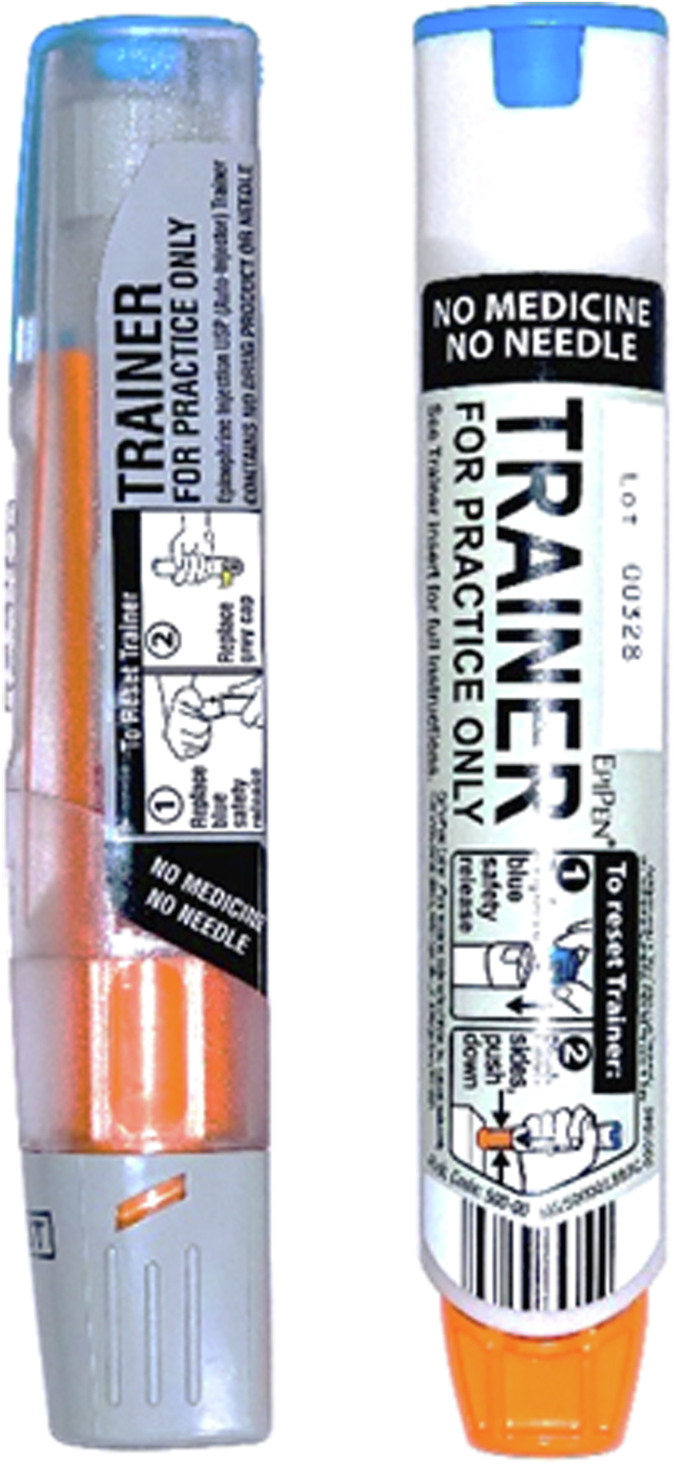
Brand and generic EAI trainers used in focus groups.

Beginning in step 2 of the hypothetical journey, participants were able to physically hold and manipulate both a brand EAI (EpiPen) and a generic EAI trainer device (no drug or needle contained in either) as they considered their potential reactions in the scenario. For the virtual groups, the moderator asked participants to open a sealed package that had been mailed to them, which contained the brand EAI and generic EAI trainer devices and a printout of the journey-mapping exercise (which was also shown on screen). This hands-on comparison facilitated the discussion about potential questions and barriers (Fig 1). At the end of the hypothetical journey, the groups concluded with a discussion summarizing the journey process that allowed participants to provide final thoughts, including their reactions to differences between brand and generic device user interfaces.

### Data collection

One experienced moderator and one notetaker attended each in-person and virtual focus group, and other team members observed remotely. Each focus group lasted approximately 90 minutes and was audio recorded and transcribed. RTI International’s Institutional Review Board approved the study. Informed consent (adults) or assent (adolescents) was provided by all participants. Parents or guardians of adolescents provided permission for their child to participate. Participants received $125 reimbursement for their time.

### Data analysis

To identify trends, the research team conducted the coding and analysis in phases consistent with grounded theory in qualitative analysis.^18^^,^^19^ NVivo qualitative data analysis software was used to aid the analysis (QSR International, v12). Two team members coded the transcripts via thematic analysis. Half of the transcripts were selected for double coding to establish interrater reliability. After interrater reliability was established, the remaining transcripts were coded independently.

## Results

### Participant characteristics

Fifty participants (11 adolescent users, 18 adult users, 21 adult caregivers) took part in the 8 focus group sessions. Table II shows participant characteristics.

**Table II:** Characteristics of 50 focus group participants

Characteristic	Caregiver of EpiPen user (n = 21)	Adult EpiPen user (n = 18)	Adolescent EpiPen user (n = 11)
Format			
In person	15 (71)	6 (33)	5 (45)
Virtual	6 (29)	12 (67)	6 (55)
Sex			
Male	5 (24)	9 (50)	4 (36)
Female	16 (76)	9 (50)	6 (55)
Prefer not to answer	0	0	1 (9)
Race/ethnicity			
White (non-Hispanic)	7 (33)	9 (50)	6 (55)
African American (non-Hispanic)	5 (24)	6 (33)	3 (27)
Asian (non-Hispanic)	0	2 (11)	2 (18)
Hispanic	2 (10)	0	0
Prefer not to answer	0	1 (6)	0
Education∗			
High school	0	2 (11)	—
Some college or technical school	4 (19)	5 (28)	—
College graduate	8 (38)	6 (33)	—
Postgraduate	9 (43)	5 (28)	—

Data are presented as nos. (%).

∗ We did not ask about education for adolescent participants because all were in middle or high school (none had graduated from high school).

On the basis of responses from the pregroup questionnaire or Zoom poll questions, most participants (46, 92%) were either somewhat or very satisfied with their current EAI, and 74% (n = 37) had received training, most from a health care provider (Table III).

**Table III:** Experience with current medical device for 50 focus group participants

Characteristic	Caregiver of EpiPen user (n = 21)	Adult EpiPen user (n = 18)	Adolescent EpiPen user (n = 11)
Satisfaction with current medical device			
Very dissatisfied	1 (5)	1 (6)	0
Somewhat dissatisfied	1 (5)	1 (6)	0
Somewhat satisfied	9 (43)	10 (56)	5 (45)
Very satisfied	10 (48)	6 (33)	6 (55)
Training			
Yes	15 (71)	13 (72)	9 (82)
No	5 (24)	5 (28)	1 (9)
Don’t remember	1 (5)	0	1 (9)
Training was provided by:∗			
Doctor	5 (33)	3 (23)	4 (44)
Nurse	9 (60)	6 (46)	2 (22)
Someone in doctor’s office	3 (20)	4 (31)	4 (44)
Pharmacist	4 (27)	2 (15)	0
Someone else	1 (7)	3 (23)	5 (56)
Current device			
EpiPen/EpiPen Jr	14 (67)	15 (83)	8 (73)
Epi Injection USP	3 (14)	3 (17)	3 (27)
Unclear whether brand or generic EpiPen†	7 (33)	—	—

Data are presented as nos. (%).

∗ Participants could select more than 1 response.

† For 7 participants from the in-person focus group, it was unclear from screener data whether they used both brand and generic EpiPens, or just the brand or generic.

### Participant thoughts on generic drugs

When asked to share thoughts or feelings about the term “generic drug,” participants in all groups mentioned a perceived financial benefit of generic drugs compared to brand drugs. Other responses consistent across audience segments and the in-person and virtual groups included the following: noted similarities between generic and brand medications, comments on the overall availability of generics, questions about how efficacy or safety of a generic drug compares with its brand counterpart, the influence of insurance coverage on costs and availability, and comments about how ingredients in a generic drug compare with those in the brand drug (same medication/ingredients vs close but not exactly the same). Table IV lists the most frequently listed phrases, with similar responses grouped together. Some themes were mentioned only in caregiver groups, including the following: (1) preference for brand over generic drugs, (2) questions about whether generics have the same FDA approval and oversight as brand drugs, (3) the overall appearance of the generic drugs, and (4) trusting the pharmacist’s decision about generic drug substitution.

**Table IV:** Most frequent responses to thoughts or feelings about generic drugs

Item group	Similarly grouped phrases used by participants	Caregivers (n = 3)		Adult users (n = 3)		Adolescent users (n = 2)		Total no. of groups
		In person (n = 2)	Virtual (n = 1)	In person (n = 1)	Virtual (n = 2)	In person (n = 1)	Virtual (n = 1)	
Financial impact or out-of-pocket cost	Cheaper, affordable, lower cost, savings, less expensive, inexpensive, cost-effective (for some people), able to save money	2	1	1	2	1	1	8
Similarities between medications	Serves the same purpose, works the same, somewhat the same, not identical, same quality as name brand, same drug, same medication	2		1	2		1	6
Availability	Easier to get, more readily available, more available, patent has passed∗	2		1	1	1		5
Question efficacy/safety	Does it work the same? Good enough? Might not work as well; I question the potency; device may not be as easy to use; more side effects	2	1	1	1			5
Insurance	Insurance required, insurance coverage, will medical assistance cover it?	2	1		1			4
Ingredients	Check ingredients, different fillers, same medication/ingredients, small changes, close but not exact	2	1	1				4
Naming conventions	Name-brand recall, confusing/different names, long name, less marketing so poor associations with brand name	2		1				3
Brand/generic preference	Prefer name-brand Rx, gravitate toward generic, [preference] depends on the drug/issue, [generic is] least favorable	2	1					3
Less effective	Less effective, lesser version, lower quality, may be less effective, possibly not as effective as medication [I’m] currently using		1		1	1		3
Quality	Inferior product, subpar, generics aren’t as good as the brand drugs		1		1		1	3
FDA approval/oversight	Made in USA? Tested by FDA?	2						2
Generic design	Can look different; boring, bland packaging	2						2
Defer to pharmacist	Trust pharmacist with generic prescription	2						2
OTC or store brands	OTC meds, a store brand, or something not having an actual name				1		1	2

*OTC,* Over the counter; *Rx,* prescription.

∗ “Patent had passed” was interpreted as expiration of patent on brand product.

### Journey mapping step 1: “It is time to get a refill for the drug device”

In step 1, participants described their thought process when they need a refill for their EAI. This step was used only to frame the topic for participants and to set them up for the remaining journey mapping steps; therefore, results were not summarized.

### Journey mapping step 2: “The refill received is for a generic drug device instead of your regular drug device”

In step 2, participants were asked to imagine a situation in which they receive their medicine refill from the pharmacist, but the EAI product is a generic version of their usual brand EAI instead of the EAI brand they typically receive.

#### Step 2: Reactions

Table V includes a summary of participants’ emotional and other anticipated reactions, along with sample quotes illustrating the theme. Participants across in-person and virtual groups described both positive and negative anticipated thoughts or feelings when faced with an unexpected substitution of a generic version of their EAI. At least 1 participant in each of the groups described an anticipated negative reaction. The most frequent negative reactions related to confusion about why they received a different EAI product than expected or feelings of doubt and anxiety, particularly related to the generic product’s quality or their ability to use the device effectively in an emergency. In more than half of the groups, participants expressed anger and frustration about being switched to a generic or not being consulted about the product change before the product was dispensed.

**Table V:** Journey mapping step 2: Receiving a generic autoinjector—Reactions and emotions

Reaction	No. of groups mentioning reaction		Exemplary quotes
	In person (n = 4)	Virtual (n = 4)	
Negative	4	4	
Confusion	2	4	• I would be confused because I’m expecting one thing, and I’m getting another, and nobody told me. (Adult user, In person-3) • [My reactions were] “oh boy” and “ugh.” Because now I’m like, “OK. What do I have new? What do I have to learn that’s new and is there going to be a transition for it? Why did we get the generic?” (Caregiver, Virtual-3)
Doubt or anxiety	2	4	• Yeah, I’d be worried if they built the device cheaper, like used crappy plastic. I’d be worried if I might break it, or I’d just be worried about what kind of stuff they put into it would be cheap and crappy. (Adolescent, In person-2) • In the heat of the moment, I would forget which one to do first: take off the blue or unscrew the bottom. The 2 steps. I’m just trying to save my kid’s life. I can’t remember which one comes first. That’s my biggest concern. (Caregiver, Virtual-3)
Anger/frustration about switching or not being told about the switch	3	2	• That I was really angry. Sorry I wrote “pissed.” Why was I given generic if I thought I was going to get an EpiPen? . . . I want to see a study. I want to see the results. (Caregiver, In person-1) • Well, I don’t want anyone else making that decision for me. So, I want a choice. If there is a generic, or a brand, I want to make that decision. I don’t want someone else making that for me. (Adult user, In person-3) • I would be annoyed if I found out the insurance company gave me generic just to save themselves some money. (Adult user, Virtual-1)
Disappointment	1	1	• Disappointed yeah, it’s like you go to the Cadillac dealership, and instead of a Cadillac you pull around in a Toyota Corolla. . . . But then afterwards, I would feel relieved because I know that’s going to be cheaper. So my initial would be disappointed, it’s like, “Aw man.” (Adult user, In person-3) • I’m not happy about it, but as long as it works, I’m fine. I’m not happy about the fact that they have to change these things. And you do wonder why they change it. (Adult user, Virtual-2)
Distrust in a person or organization	2		• If it’s a random brand that I’ve never heard of before, and it’s for something lifesaving, I wouldn’t really trust it. (Adolescent, In person-2)
Distrust in medication or device		1	• [I] worry about actually getting the proper dosage. (Adult user, Virtual-1)
Positive	3	3	
Happy, grateful or relieved	3	1	• At the end of the day, I’m just happy to know that there’s something there in case my kid does need anything. (Caregiver, Virtual-3)
Trust in doctor	1		• If my doctor told me it was safer, even though it is more difficult to use, if it’s safer for my young child, if it was safer for 2 years and younger, then I would have no questions asked. I would say, OK. I’ll take it. (Caregiver, In person-1)
Trust in medication or device		3	• I mean it works. I’m just assuming that it’s the same thing as what I’m taking. So it doesn’t really matter. . . . Epinephrine is epinephrine no matter what injector it’s in. It’s still epinephrine. (Adult user, Virtual-1)
Neutral	4	2	
Curious or questioning	3	2	• The initial concern, why is there a shortage? What are the differences because now it’s forcing me to think, which a lot of people don’t want to do depending on the time and the circumstance. That’s about it. (Caregiver, In person-1) • It’s just because it’s not what we’re used to. Of course, they’re going to have questions. (Adolescent, In person-2)
Other neutral	2	1	• I wrote down “no big deal.” (Adult user, Virtual-1)

In three quarters of the groups, participants anticipated positive feelings. Most frequently, participants said they would be happy, grateful, or relieved to have a less expensive treatment option and to have the medication available to treat a severe allergic reaction.

In three quarters of the groups, participants also identified more neutral reactions, such as the following: general acceptance of the generic substitution, curiosity about the reason for the switch, and questions about how the generic device differs from the brand device in efficacy and functionality.

#### Step 2: Questions, anticipated challenges, and information sources

Questions and anticipated challenges in making the switch from the brand EAI to the generic EAI focused on the efficacy, cost, ingredients, dosage, and functionality of the generic EAI and how it would compare with their brand EAI. Table VI provides themes and exemplary quotes.

**Table VI:** Themes and exemplary quotes from journey-mapping exercise

Journey mapping step	Theme	Exemplary quotes
Step 2: Questions, anticipated challenges, and information sources when receiving a generic device	Functionality	• If I’m going to have to use [the generic] going forward, I’m going to have to relearn the steps that I take to use my EpiPen. . . . It’s something I’ll have to get used to, but it isn’t terribly bad to where I hate this device or dislike it at all. (Adult user, Virtual-2)
	Effectiveness	• Is it going to work? Is it going to have the same ingredients? . . . Contents of the original EpiPen? That’s going to be my first thought. (Caregiver, In person-1)
	Quality	• The branded has less packaging, it seems like, or materials than [the generic]. So would the cost of the branded one be cheaper than the generic one? (Adult user, Virtual-1)
Step 3: Questions, anticipated challenges, and information sources when using the generic device	Functionality	• In the back of my mind, I’m panicking. So, am I going to remember to twist and pull, or do I just go ahead and pretend I’m using the [brand AI]? (Caregiver, In person-1) • [The generic device] is more difficult for people with dexterity issues. . . . If they’re an older person, they have arthritis . . . it’s going to be more difficult. This twist-off, this cap, versus nothing [on the brand device] . . . for people with the dexterity issues, that could be an issue. (Adult user, Virtual-2)
	Effectiveness	• Is it going to be more effective? Is it going to be working? (Caregiver, In person-1) • I think my first thoughts would be, will this really work, or is it going to give me the same feeling . . . as the brand one? (Adolescent, Virtual-4)
	Quality	• Where is the quality being compromised? (Adult user, In person-3) • Being a generic, it’s probably going to have less bells and whistles on it. It’s just a different . . . carrier for the medication, but . . . it’s probably going to . . . be a little bit more complicated and maybe on the cheaper side. (Adult user, Virtual-1) • I don’t want something bad to happen. If I need it, will it break or something like that? (Adolescents, Virtual-4)
Reactions to differences in user interface	Design	• [The generic AI] is a little harder to grasp. Because it’s chunkier. (Adolescent, In person-2) • I kind of like this bigger [generic] one because I’m used to holding a lot of bigger stuff so it makes it a little bit easier for me. (Caregiver, In person-1) • This outer shape case on the generic, it reminds me of the carrying case that the name brand comes in. It seems like the generic doesn’t have an exterior case. And that I do really like. It makes me feel more confident in being able to remove it in time. (Adult user, Virtual-1)
	Functionality	• The twist-off part [on the generic AI] is no good. Because what happens if you’re the only one around and you’re not able to twist it off? (Adolescent, In person-2) • [The brand AI] is one less step, but [the extra step of twisting in the generic AI] is the stability I want. (Caregiver, In person-1) • I also think that the [generic AI] is a 2-step process. The branded is one step, pop off the blue, bam, ready to go. With the [generic], I got to twist off the bottom, then I got to pull the blue off, then I’m ready to go. It’s one extra step, and in emergencies you don’t need extra steps. (Adult user, Virtual-2)

*AI,* Autoinjector.

Participants across all groups said that they would ask a pharmacist or health care provider for more information or answers to their questions. In 7 of the 8 groups, some participants mentioned using online resources (eg, YouTube, manufacturer’s website, general online search) to find the information they needed. Participants in 3 groups identified product package inserts as a source of additional information.

### Journey mapping step 3: “It’s time to use the generic device for the first time”

In step 3, participants were asked how they would approach using the generic EAI for the first time.

#### Step 3: Reactions

Table VII summarizes participants’ emotional and other reactions along with sample quotes illustrating the theme. Participants across in-person and virtual groups anticipated both positive and negative thoughts and feelings associated with using the new generic EAI for the first time. At least 1 participant in each group described an anticipated negative reaction, most often related to doubt or anxiety about using the device correctly and whether they would successfully administer an effective dose in a potentially life-threatening situation. In half of the groups, participants expressed distrust that the generic device, or the medicine in it, would be as effective as their brand device.

**Table VII:** Journey mapping step 3: Using a generic autoinjector—Reactions and emotions

Reaction	No. of groups that mentioned reaction		Exemplary quotes
	In person (n = 4)	Virtual (n = 4)	
Negative	4	4	
Doubt or anxiety	4	4	• First time trying it—scared, worried, frightened. (Adult user, In person-3) • I wrote “scared but trying to remain calm.” (Caregiver, In person-1) • A little worried, yeah . . . because there’s no preparation before you use it. It’s just kind of like . . . you have to go with it. (Adolescent, In person-2)
Distrust in medicine or device	2	2	• I have a very negative connotation with it [generics]. I think of generic, I think cheaper, and with a lifesaving device that I might really need, I don’t want it to be cheaper. (Adolescent, In person-2) • When I’m actually needing it, I would probably go back to my 70s way of thinking . . . that “oh my goodness, it’s cheaper, so it has to be less effective.” (Adult user, In person-3)
Frustration with not having familiar device		1	• Let’s just say I take the blue part off [of the generic device] first and then twist it. Is it going to still work if I do it that order? . . . It’s telling your brain to do the opposite of the name brand. (Caregiver, Virtual-3)
Confusion		1	• What step comes first? I can’t remember. (Caregiver, Virtual-3)
Positive	4	4	
Trust in medication/device	3	3	• I’m trusting just overall. With either one [generic or branded] that I would use. (Caregiver, In person-1)
Grateful or relieved		2	• I would say that I’m glad I have it. And I would be proud of myself for using it and deciding to have it and take care of myself and my health. (Adult user, Virtual-2)
Hopeful	2		• I hope it works. (Caregiver, In person-1)
Trust in myself		1	• I would feel somewhat confident just because I practiced with it. (Caregiver, Virtual-3)
Neutral	2	2	
Curious or questioning		1	• I mean, the feel was different, the steps were different. So is it going to work the same? (Adult user, Virtual-2)
Other neutral	1	1	• It’s mind over matter. You’ve got it, you’ve learned how to use it, you got no other choice. (Adult user, In person-3)

Across all groups, participants also anticipated positive feelings about using the generic EAI. At least 1 participant in three quarters of the groups said they would trust the medication or device to work effectively. In half of the in-person groups, participants expressed hope or optimism that the generic product would be effective, and in half of the virtual groups, participants noted they would feel grateful or relieved to have the generic EAI available when they needed it to relieve their symptoms.

Participants in half of the groups mentioned more neutral reactions, including curiosity about differences between the generic and brand devices and general acceptance of which version of the EAI was available, especially in an emergency.

#### Step 3: Questions, anticipated challenges, and information sources

Most frequently, participants mentioned questions and challenges centered around remembering how to use the new generic device and its functionality. Some participants mentioned the importance of having a trainer device, because the ability to practice provides confidence they will be able to use the EAI correctly when needed. Other comments focused on the generic EAI’s effectiveness and quality. Table VII provides themes and exemplary quotes.

To get answers about how to use the brand and generic EAIs, participants most frequently said that they would turn to demonstration videos (eg, available on YouTube or the manufacturer’s website) or in-person (live) training from a health care provider or pharmacist. Participants in a few groups said that they would review the patient package insert.

### Reactions to differences in user interface

After holding and manipulating the actual brand and generic EAI trainer devices, participants shared their thoughts on similarities and differences. Most frequently, comments related to differences in the look and feel of the device user interface (eg, size) or the functionality and design (particularly related to the generic’s twist-off cap and number of use steps). While most comments indicated a preference for the more familiar brand EAI device, some participants pointed out aspects of the generic device that they thought were better or equal to the brand device.

## Discussion

This study was conducted as part of FDA’s ongoing generic drug development research program, which increases publicly available data to support development of complex, generic drugs and DDCPs. For generic EAIs, approval of multiple generics will likely reduce product cost significantly and may enhance compliance with international recommendations that patients with severe allergic reactions always carry 2 unexpired EAIs and administer epinephrine early when a severe allergic reaction occurs.^2^ Currently, the cost of the generic Teva EAI and the authorized generic EAI have average acquisition costs are about half that of the EpiPen‖ and generic cost savings are generally not optimized until 3 or more generic versions of a product enter the market.^20^

This study’s focus group discussions and journey-mapping exercises revealed how differences in device design and user interface features of the generic EAI versus the EpiPen affected participants’ views of product quality, efficacy, and device usability. Responses from in-person and virtual focus group participants shared similar themes.

General thoughts about generic drugs ranged from perceptions among adolescent patients that the generic drug might not be as good as the brand to feedback from some adult patients and caregivers that generics offer lower cost and increased availability at the pharmacy. Some participants noted that generic drugs are essentially the “same thing” as the brand drug, yet other participants wondered whether generic drugs work as well as the brand or if the generic could have additional quality or safety issues. This highlights lingering uncertainty about generic drugs and generic substitution for a brand EAI in this sample.

During the journey-mapping exercise, participants expressed a variety of anticipated positive and negative reactions to generic substitution of their EAI. Positive reactions focused on potential cost savings, optimism that the generic would perform as well as the EpiPen, and appreciation that the EAI was available if needed in an emergency. The most common negative reaction was confusion about why they received a different EAI than expected. Some participants felt angry or frustrated about not being consulted/informed about the change ahead of time or curious about why generic substitution occurred. For some, receipt of a different product produced feelings of doubt and anxiety about the generic product’s quality or about their ability to use the device effectively in an emergency. Some participants raised questions about differences between the generic and EpiPen devices: Does it work as well? Does it function differently?

These results suggest that patients and caregivers who use EAIs may appreciate and benefit from discussions with their prescribers about generic EAIs and generic substitution.# The cost savings associated with generic EAI substitution are appealing to patients but do not eliminate their concerns. Therefore, setting expectations and explaining that a generic EAI will have the same safety and clinical effect as the brand EAI may help minimize the anxiety, frustration, and confusion some patients and caregivers expressed in this study when faced with an EAI generic substitution scenario. These anticipated reactions may be layered on top of other preexisting worries among patients with severe allergic reactions and their caregivers about when to use the EAI, potential side effects, needle phobias, and the force required to inject the drug.^21^

The results of this study showed many similarities to results from a similar journey-mapping focus group study conducted in 2019-20 among adult and adolescent users of a dry powder inhaler. Although the dry powder inhaler was a daily-use medication for managing a chronic disease (asthma or chronic obstructive pulmonary disease), the generic substitution scenario raised similar questions among participants and anticipatory anxiety about product performance and the ability to use the generic correctly.^22^

As participants manipulated the EpiPen and generic trainer devices, they compared and contrasted device designs. Participant feedback most often related to differences in look and feel (eg, size, force to execute user tasks, hand grip) or functionality and physical design differences (eg, EpiPen has a carry case, but the generic does not; generic has a twist-off cap at the needle end, and EpiPen does not). Some participants mentioned the importance of having a trainer device, because the ability to practice using the trainer builds confidence that they will be able to use the EAI in an emergency.

To obtain FDA approval of a generic DDCP, the firm must demonstrate, among other things, that differences between the generic and brand product device designs will not increase the risk for user errors and that the generic will have the same clinical effect and safety profile as the RLD when generic substitution occurs without additional training from prescriber or pharmacist. The generic EAI from Teva Pharmaceuticals met these criteria for approval; however, as focus group outcomes show, health care providers can help prepare patients and caregivers for generic substitution by setting expectations that the generic will be highly similar to the brand product and will have the same safety and clinical effect. Additionally, different individuals may prefer different design features over others—a couple of focus group participants preferred aspects of the generic EAI design.

Although the focus groups provided a great deal of rich data, this research had several limitations. We experienced an unanticipated 2-year gap between conducting the in-person and remote focus groups, so we chose to report findings separately for these 2 study phases and highlight key differences. Nonetheless, many of the themes held together across and within all the focus group interviews, suggesting theoretical saturation of data collection. Like all qualitative research studies using focus groups, the findings of this study may not represent the views of larger population segments. To optimize feedback about switching from EpiPen to a generic EAI, recruitment processes ensured that we heard perspectives from 3 distinct user groups: adult patients, adolescent patients, and caregivers. However, our participants and their shared perceptions and attitudes represent only a “snapshot” of the entire EAI user population.

### Conclusion

Published studies show that the American public’s perceptions of and knowledge about generic drugs have improved over the past 20 years; however, these focus groups revealed lingering uncertainties about how differences between a generic EAI and the EpiPen would affect users’ ability to use the generic in an emergency and how well it would work. While patients and caregivers are interested in the potential cost savings and increased access to treatment offered by generic EAIs, they want to be informed by their prescriber or pharmacist about generic substitution before or when it occurs. Outcomes from these focus groups suggest opportunities for FDA, health care professional organizations, and patient advocacy organizations to improve generic drug literacy among adults, adolescents, and health care providers.

Educating the allergy/clinical immunology provider community about generic DDCPs and developing patient-focused resources and tools for use in the clinical practice setting could improve adherence to timely treatment of severe allergic reactions with generic epinephrine autoinjectors. Health care providers, patients, and caregivers should know that a generic version of a given brand epinephrine autoinjector product will have (1) the same safety and clinical effect as the brand product and (2) a highly similar (although not identical) device. Patients with a history of severe allergic reactions and their caregivers should be (1) informed when generic versions of their EAI are available and may be substituted for their brand product at the pharmacy, (2) reassured that the generic product will perform as well as the brand product, and (3) encouraged to practice with the generic product trainer so they feel prepared to use the product in an emergency. FDA provides several resources about similarities and differences between brand and generic products that are available in different formats such as Q&A handouts about generic drugs, patient education handouts, brochures, fact sheets, infographics, and public service announcements., which can be accessed at https://www.fda.gov/drugs/generic-drugs/patient-education.

## Disclosure statement

Funded by contract HHSF223210810113C from the Office of Research and Standards/Office of Generic Drugs, US 10.13039/100009210Food and Drug Administration.

Disclosure of potential conflict of interest: The authors declare that they have no relevant conflicts of interest.

The views expressed in this article are from the authors and do not necessarily reflect the official policies of the Department of Health and Human Services, the National Institutes of Health, and/or the Food and Drug Administration; nor does any mention of trade names, commercial practices, or organizations imply endorsement by the US government.

## Footnotes

∗ While epinephrine autoinjector products approved by the FDA are indicated for intramuscular or subcutaneous injection, Lieberman et al noted in 2015 that there are no studies evaluating the pharmacokinetics of a subcutaneous injection of epinephrine in the lateral thigh.^1^

† The Joint Task Force on Practice Parameters represents the American Academy of Allergy, Asthma & Immunology; the American College of Allergy, Asthma & Immunology; and the Joint Council of Allergy, Asthma & Immunology.

‡ EAIs meet the regulatory definition of a combination product because they contain both a drug (epinephrine) and a device (the autoinjector).^4^

§ Generic product cost savings generally are not optimized until 3 or 4 generic versions of a brand product enter the market.^5^

¶ The terms “name brand” and “brand” are often applied to the innovator drug. A generic drug is “the same as” a particular innovator drug in defined ways (Table I). However, some generic drugs also have brand names.

## Footnotes

‖ IQVIA National Sales Perspective; search performed March 21, 2023.

# Laws about whether generic substitution can occur without advanced prescriber or patient notification vary by state.
