# Supplementary material for: Generic substitution of epinephrine autoinjectors: Patient and caregiver perceptions and attitudes
Source: J Allergy Clin Immunol Glob. 2023 Sep 14;3(1):100170. doi: 10.1016/j.jacig.2023.100170 (PMC10590739; doi:10.1016/j.jacig.2023.100170)
Supplement: Supplementary Tables [file mmc1.docx]

**Online Repository Tables**

**Table A1. Description of Eligibility**

| - Within each group, the recruitment strategy targeted a distribution of participants across age groups, sex, and education levels (for adult and caregiver groups only). - Eligible participants for both the in-person and virtual groups were adults, adult caregivers, and adolescents who either personally use (adults and adolescents) or care for someone who uses (caregivers) the EpiPen, EpiPen Jr, or Ephedrine Injection USP brand of AI and could speak and read English fluently. - We excluded individuals who work in the healthcare, marketing, advertising, or pharmaceutical industries and people who work for the Department of Health and Human Services, because their knowledge and experiences may not reflect those of the average consumer. - For the four in-person groups, to achieve geographic diversity, we collected data through four focus groups in two cities in different parts of the country (Minneapolis, MN, and Atlanta, GA). - Eligible participants for the four virtual focus groups were recruited from all over the United States and were required to have a high-speed internet connection, have a laptop/computer with a webcam and audio, and be able to receive a package of focus group materials in the mail. - To keep groups a manageable size, each in-person focus group included up to 10 participants, and each virtual focus group included up to six participants. - We overrecruited by three participants to account for no-shows. |
| --- |

**Table B1. Focus Group Topics**

| **Topics** |
| --- |
| 1. Survey/poll questions about experience with current brand of AI |
| 1. Participants’ current experiences with their drug device |
| 1. Overall perceptions of generic drugs |
| 1. Journey Mapping Exercise. At each step, participants were asked to describe their thoughts, feelings, and other reactions. Participants were also asked what questions they would ask, the challenges they would face, how they would get information, and the actions they would take at each step. |
| 1. Reactions to Differences in User Interface |

**Table B2. Journey Mapping Handout**

|  | **Step 1 – STARTING POINT:**  It is time to get a refill for the drug device | **Step 2 – SECOND POINT:**  The refill received is for a generic drug device instead of your regular drug device | **STEP 3 – THIRD POINT:**  It’s time to use the generic drug device for the first time |
| --- | --- | --- | --- |
| **Thoughts**  What is the first thing that comes to your mind? What questions do you have? What information would you like to have? |  |  |  |
| **Actions**  What tasks are involved with this step?  What actions will you take? |  |  |  |
| **Challenges**  What would make things easier during this step?  What do you worry about? |  |  |  |
| **Feelings**  What are you feeling? Write any positive words above the line and any negative feelings below the line | **------------------------------------------------------** | **--------------------------------------------------------** | **-------------------------------------------------------** |
